# The challenges and potential solutions of achieving meaningful consent amongst research participants in northern Thailand: a qualitative study

**DOI:** 10.1186/s12910-023-00991-0

**Published:** 2023-12-19

**Authors:** Rachel C Greer, Nipaphan Kanthawang, Jennifer Roest, Carlo Perrone, Tri Wangrangsimakul, Michael Parker, Maureen Kelley, Phaik Yeong Cheah

**Affiliations:** 1 Mahidol Oxford Tropical Medicine Research Unit, Faculty of Tropical Medicine, Mahidol University, Bangkok, Thailand; 2 The Ethox Centre, Nuffield Department of Population Health, University of Oxford, Oxford, UK; 3 Wellcome Centre for Ethics & Humanities, Nuffield Department of Population Health, University of Oxford, Oxford, UK; 4 Centre for Tropical Medicine and Global Health, Nuffield Department of Medicine, University of Oxford, Oxford, UK

**Keywords:** Informed consent, Decision making, Literacy, Translators, Agency, Hill tribe, Ethnic minority, Thailand, Research ethics, meaningful consent

## Abstract

**Background:**

Achieving meaningful consent can be challenging, particularly in contexts of diminished literacy, yet is a vital part of participant protection in global health research.

**Method:**

We explored the challenges and potential solutions of achieving meaningful consent through a qualitative study in a predominantly hill tribe ethnic minority population in northern Thailand, a culturally distinctive population with low literacy. Semi-structured interviews were conducted with 37 respondents who had participated in scrub typhus clinical research, their family members, researchers and other key informants. A thematic analysis was conducted.

**Results:**

Our analysis identified four interrelated themes surrounding participants’ ability to give consent: varying degrees of research understanding, limitations of using informal translators, issues impacting decisions to join research, and voluntariness of consent. Suggestions for achieving more meaningful consent included the use of formal translators and community engagement with research populations.

**Conclusions:**

Participant’s agency in decision making to join research should be supported, but research information needs to be communicated to potential participants in a way that they can understand. We found that improved understanding about the study and its potential benefits and harms goes beyond literacy or translation and requires attention to social and cultural factors.

## Introduction

Thailand has a large hill tribe ethnic minority population, many of whom live in the mountainous border regions of northern Thailand. Each group has their own language, customs and beliefs. The three main groups in Chiangrai province are the Akha, Lahu and Hmong [1]. Hill tribe groups are often socially, politically and economically marginalised, which has the potential to contribute to significant health vulnerabilities. Historically they were not considered to be Thai nationals, despite many having lived in Thailand for generations, having migrated from surrounding countries, such as, China, Myanmar and Laos. In 2021, over 500,000 people were still registered as being stateless and this is likely to be an underestimate [2]. An estimated 14% of Chiangrai’s hill tribe population do not have Thai citizenship [1].

Without Thai citizenship, hill tribe members are not entitled to free healthcare, cannot own land and have restricted mobility [3–5]. They may have less access to education and limited employment opportunities with many working in daily labour jobs or agriculture [3–6]. Other vulnerabilities include social and geographical isolation, poverty and Thai language barriers [3, 4, 6, 7]. All of these challenges can affect hill tribe members’ ability to access healthcare, which can result in poorer health outcomes and measures such as vaccination coverage [7, 8]. Clinical and public health research engaging hill tribe members can be a valuable way to better understand their specific health burdens, barriers to health care, and help inform interventions that are more responsive to their needs.

In a previous paper we have described how challenges in daily living, faced by hill tribe members, can impact their experiences of taking part in research, including their perceived benefits, burdens and hidden burdens of participation [6]. In this analysis, we examine the specific challenges around consent to help inform improvements in more supportive engagement of hill tribes in health research.

Meaningful, valid consent is one of the cornerstones of ethical research, ensuring that prospective participants are given adequate information to understand the research, including the potential risks and benefits of participation [9, 10]. However, it is widely appreciated that achieving valid consent in a meaningful way, so that it is genuinely responsive to participants’ needs and vulnerabilities, as well as supportive of their autonomy and agency, henceforth referred to as ‘meaningful consent’ is not straightforward [11–13]. For instance, opinions vary on the extent and nature of information researchers should provide to participants, as well as the depth of understanding deemed adequate for participants to make informed, autonomous decisions [11, 14]. Moreover, the way people make decisions varies and is influenced by social, cultural and political factors, including the availability of alternative options for accessing healthcare. These structural factors can influence the voluntariness of people’s decision making [15, 16].

This paper explores the challenges of achieving meaningful consent for research participants in northern Thailand with a population consisting predominantly of hill tribe groups. This was identified as a key theme in our international research study - REACH: Resilience, Empowerment and Advocacy in Women’s and Children’s Health, a study which aimed to increase understanding of how vulnerability and agency should be understood in research ethics. We also offer suggestions for improving the consent processes for other marginalized populations.

### Study setting and methods

Chiangrai is a predominantly rural province in northern Thailand, which borders Myanmar to the north and Laos to the east, with all three countries meeting at the Golden Triangle. In 2020, it had a population of 1.3 million, of whom approximately 20% are from a hill tribe ethnic minority group [17, 18]. Chiangrai Province is one of the poorer provinces in Thailand with a household monthly income of 354 British Pounds (versus 620 British Pounds national average) [19]. The main occupation is agriculture.

Chiangrai Clinical Research Unit is a small satellite unit of the Bangkok-based Mahidol Oxford Tropical Medicine Research Unit (MORU), established in 1979. It is located in the provincial capital city and was established in 2015, to conduct research to answer important health questions in the province. Areas of research include scrub typhus, febrile illnesses and empirical research in global health bioethics.

### Linked scrub typhus studies

Our qualitative study drew participants from two clinical studies on scrub typhus, which is an under recognised but leading cause of undifferentiated fever in Thailand [20–22]. It is a bacterial infection, transmitted through the bite of infected mites which are commonly found in rural areas placing those living and working there at increased risk [23, 24]. Descriptions of the clinical studies are provided in Table 1 [6]. Of the two studies we looked at, one is a randomised controlled trial (RCT) evaluating the optimum treatment for scrub typhus. The other is an observational study exploring the immune response to scrub typhus infection in children and adults.

**Table 1:** Linked scrub typhus study details

Study details		
Study title	The Scrub Typhus Antibiotic Resistance Trial comparing doxycycline and azithromycin treatment modalities in areas of reported antimicrobial resistance for scrub typhus	Eschar investigations to improve diagnostics, understand early immune responses and characterize strains for vaccines in scrub typhus
Study design	Randomized controlled trial (RCT)	Observational
Aims	Determine the optimum treatment for scrub typhus by comparing three oral antibiotic treatments	Improve understanding of the immune response to scrub typhus and investigate possible early diagnostics
Study population	Patients ≥ 15 years old hospitalised with non-severe scrub typhus	• ≥ 7 years old AND • Patients presenting to hospital with scrub typhus OR • Controls with skin injuries or attending minor surgery, who have had scrub typhus in the past or live in an endemic area.
Study processes	• Randomised to 1 of 3 treatment arms • Demographic & clinical data • Blood & urine samples at enrolment • Daily clinical review while in hospital • A further 6 or 12 blood samples over the next week • Follow up at 2 and 8 weeks (clinical data, blood & urine samples)	Patients: • Demographic & clinical data • Eschar swabs, scrapings or biopsies • Lymph node aspirates from a subgroup • Blood & urine samples at enrolment • Follow up at 2 weeks (clinical data, blood & urine samples) Controls: • Demographic & clinical data • Blood & urine samples at enrolment • Skin biopsies
Study benefits	• Treatment for scrub typhus (although most would be entitled to free treatment as part of routine care) • Compensation for time and reimbursement for actual travel costs for enrolment and follow-up visits • May help to improve scrub typhus treatment in the future	• No direct benefits • Compensation for time and reimbursement for actual travel costs for enrolment and follow-up visits • May increase understanding of scrub typhus disease severity and diagnostics
ClinicalTrials.gov identifier	NCT03083197	NCT02915861

*Adapted from Greer et al. Vulnerability and agency in research participants’ daily lives and the research encounter: A qualitative case study of participants taking part in scrub typhus research in northern Thailand. PLoS One. 2023;18(1):e0280056. Epub 2023/01/26. doi*: 10.1371/journal.pone.0280056. *License CC BY* [6]

## Methods

An integrated ethics, case study design was used. The case study focused on scrub typhus research participants who had or were taking part in one of the linked clinical studies. The perspectives of their family members, researchers, ethics committee members and key community informants were included within the boundaries of the case.

Semi-structured interviews were conducted between March 2018 and June 2019. The REACH respondents were selected from three groups using purposive and snowball sampling techniques. Group 1 included female and child research participants (the focus of the REACH study) and their family members from the two clinical studies. Group 2 consisted of researchers, healthcare workers and ethics committee members who had been involved in the clinical studies. Group 3 included key community informants such as healthcare workers, local researchers and community leaders. In total, 37 respondents were recruited and six declined to participate, typically due to time constraints.

NK, a Thai research nurse and RCG, a British research physician living in Chiangrai conducted the interviews. Both interviewers are female, familiar with the local context and have received qualitative methods training. NK met most respondents prior to the interview day in order to discuss participation in REACH. Several Group 2 respondents work for MORU, the same research unit as NK and RCG.

The interviews took place at a convenient location for the respondents (their homes, workplaces and local health facilities) and lasted 80 min on average. Interviews were conducted in central or northern Thai dialects or English. If the respondents were not fluent in these languages, trained hill tribe (Akha and Lahu) translators were used. The translators were experienced and received additional training with regards to the interviews, including explanations about the project and discussions of the interview guides. All interviews were recorded, transcribed verbatim and translated into English as required. Interview guides [6], were created and piloted for each respondent group. In relation to the findings reported in this manuscript, Group 1 respondents were asked about their experiences of participating in the clinical studies, including their recruitment, consent and understanding of the research. Group 2 and 3 participants were asked about the ethical challenges of conducting research in this setting, their experiences of the consent process and their perceptions of participants’ understanding. Debriefings were carried out after each interview between NK and RCG, and other team members on occasions. Interviews were conducted until data saturation was met [6].

### Analysis

A thematic analysis was conducted from a realist epistemological stance [25, 26]. Inductive themes were sought from the data during open-coding and regular team reflection sessions. This led to the creation of a coding tree which was applied and adapted on an iterative basis. NK and RCG coded all transcripts using NVivo Pro 11. Any coding inconsistencies were discussed and agreement was reached. Codes and themes were also discussed with the wider study team. A descriptive narrative approach is used to present the results [6].

## Results

Semi-structured interviews were undertaken with 37 respondents; five, second interviews were conducted, including one dyadic interview (Table 2). Most Group 1 respondents belonged to a hill tribe group (16/19, 84%), commonly from the Akha and Lahu groups. The majority were Thai citizens (16/19, 84%), two had the right to remain in Thailand and one had no legal status. A third of the Group 1 respondents needed an interpreter for the interviews and half had no formal education [6]. The ages of the children interviewed ranged from 8 to 17 years old.

We identified four interrelated themes regarding the challenges of achieving meaningful consent: (1) varying degrees of research understanding, (2) limitations of using informal translators, (3) decision making to join research, and (4) factors influencing voluntariness of consent. We also explored suggestions for improvement. Illustrative quotes are included where relevant and are referenced by the respondent group and number, and the type of clinical study (RCT or observational if relevant). Use of interpreters during the REACH interview is noted.

**Table 2:** Breakdown of REACH respondents

Respondents (number)	Interviews (number)	Other interviews (number)
Group 1: Research participants & their family members from the linked scrub typhus studies (19)	Research participants (14) Family members (5)	Second interviews (4: 3 with research participants, 1 with a family member)
Group 2: Research staff from the linked scrub typhus studies (9)	Research nurses (3) Senior research physicians (2) Hospital nurses (2) Ethics committee members (2)	Dyadic interview with 2 research nurses (1)
Group 3: Key community informants (9)	Primary care nurses (3, 1 is also a research nurse) Research nurse (1) Doctors and researchers (2) Village Chief (1) Director of a non-profit organisation (1) Informal translator & ex-village health volunteer (1)	
Total = 37		

*Reprinted from Greer et al. Vulnerability and agency in research participants’ daily lives and the research encounter: A qualitative case study of participants taking part in scrub typhus research in northern Thailand. PLoS One. 2023;18(1):e0280056. Epub 2023/01/26. doi*: 10.1371/journal.pone.0280056. *License CC BY* [6]

### Varying degrees of research understanding

The Group 1 respondents had varying degrees of understanding regarding the study and their participation in it. The vast majority knew they had joined a research study and were aware of the research processes, for example, having blood tests and attending follow-up appointments. A smaller number understood they were able to join the project because they had scrub typhus. This was supported by the two research nurses in the dyadic interview who summarised that:

*‘[Understanding] depends on the knowledge of each patient because some people might not have studied…their understanding might be limited, might have only understood … what [they] had to do but may not have understood thoroughly and profoundly what the purposes of this project are and what’s to be done*. (Research nurse 29, dyadic interview) *But most that [I’ve] come across… they’ll understand the process of joining this project as to what will be done to them, such as drawing blood, taking medicine, recording [body] temperature… as for the steps of this process they do understand – but what [they] won’t equally understand, or don’t… don’t… receive equal details will mostly be about the knowledge of the disease [itself], about… the background of the disease, things like this*…*’* (Research nurse 01, dyadic interview).

Many of the other study details were not known by the respondents at the time of our interviews, such as the process of randomisation and the specific aims of the research. However, several respondents said the research was to improve treatment and some thought the research would benefit others in the future: *‘They would publish [about it] … get the medicine and apply treatments to make it [scrub typhus] go away. To keep it - this bug, away from biting us.’* (Scrub typhus observational study participant 10).

*‘[The researchers] would come to do a study in order to help other people [so that] when they fall ill [they] would know what methods of treatment there would be.’* (Scrub typhus RCT participant 03).

On several occasions, despite having received an explanation, Group 1 respondents reported that they had limited understanding:

*‘She [participant 16] understands that… she’s carrying a disease… that’s why she is able to join [the study]… People explained to her but she didn’t understand… She tended to not understand.’* (Scrub typhus RCT participant 16, speaking through an interpreter).

Surprisingly, despite not understanding or having limited understanding several described still joining the study:

*‘She said even though they explained, she still didn’t know. She would still agree [to join the research] anyway.’* (Scrub typhus RCT participant 13, speaking through an interpreter).

#### Factors impacting understanding

Levels of research understanding were affected by factors relating to the participants and researchers. According to all respondent groups, those who had a formal education, were proficient in Thai language and were familiar with research seemed to more easily understand information about the study and their participation in research. All groups commented on the challenges for hill tribe members or migrants who did not speak Thai fluently and were reliant on translators.

*‘In the aspect of communication, and knowledge and understanding, if the patients are highly educated, they will understand us easily. But if they are of lower level [of education]… no matter how we explain they won’t understand, just know that the doctor asked them join this research. Giving some information. They will have no more doubts [questions], just sign-sign it like this. They will not have true understanding about that research*.’ (Healthcare worker 27).

*‘Those who have trouble in communication [and] language, we’re not 100% sure [about] the feedback, whether they’ll understand the things we say through an interpreter… or not… If they would 100% understand, as much as a person whom we speak to in the same language would or not.’* (Research nurse 29).

Participants’ understanding could also be affected by their perceptions and experiences of research; for many this was their first experience of research and often their first hospital admission. The majority (apart from participants from the control group in the observational study) were unwell with scrub typhus at the time of recruitment in hospital, one participant described feeling ‘drowsy’ and that this affected her memory. The potential stress of participants or their children being ill has previously been reported to affect people’s ability to understand and give meaningful (parental) consent [27, 28].

Other factors that appeared to influence participants’ understanding related to the researchers: their communication skills, choice of words, the volume of information shared and the documents used. Despite Group 1 respondents reporting that elements of the study were explained to them step-by-step, some participants seemed overwhelmed by the amount of information shared and were only able to grasp the main points:

*‘Interviewer: What did the nurse explain to you?*

*12: Explained everything… There’s a lot of things. I can’t remember anymore [laughs].’* (Mother of a scrub typhus RCT participant 12).

Rather than using the formal Thai word for ‘research’ the research nurses tended to refer to the ‘project’ or ‘study’ to try to aid participants’ understanding, although this may have blurred the distinction between research and healthcare. There is no direct translation for the word ‘research’ in Akha or Lahu languages making the concept more challenging to translate and comprehend. There was also overlap between the RCT investigating the optimum treatment for scrub typhus and their medical care for scrub typhus.

### Limitations of using informal translators

Translators are required for those trial participants who do not speak Thai. Typically hill tribe members or others unable to speak Thai fluently attended hospital with a relative who acted as an informal translator; formal translators are not routinely available. This had both positive and negative aspects. Translators enabled participants to understand more about their illness, the research and to ask questions of the staff. They could also provide support and guidance to participants.

*‘She [mother of participant 19] said she couldn’t understand while they were talking in Thai. But once a translator came to translate, she understood, she was able to see the picture.’* (Mother of a scrub typhus observational study participant 19, speaking through an interpreter).

However, the quality and extent of translation varied. Informal translators do not receive any training or support and are reliant on their own understanding and knowledge to translate complex medical terms and research terminology. One sister-in-law explained how she translated as much as she could understand:

*‘She [sister-in-law of participant 14] was, merely able to grasp the main points [of it] that shortly the treatment would take place, [by] this team, in this and that manner, with these medications being involved… There were certain words, there were many, many words that were, like, in some parts she understood [while] in some others she didn’t understand, so she felt puzzled.’* (Sister-in-law of a scrub typhus RCT participant 14, speaking through an interpreter).

Important details could be lost in translation, for example the sister-in-law who translated knew that the research was voluntary but the Group 1 respondent she translated for did not. Other translators summarised the information shared as being ‘good’ and recommended that people should join the research or simply that they needed to provide a thumbprint on the consent form without explanation of what that meant:

*‘[The participant’s niece and translator] didn’t tell anything. She said it was good. Go ahead and participate. That was it so she [participant 13] joined.’* (Scrub typhus RCT participant 13, speaking through an interpreter).

*‘She [participant 17] only knew that she had to provide a thumbprint. She didn’t know what the project was about…her translator [a neighbour] told her to provide a thumbprint so she just did. He didn’t explain to her about joining project this or that.’* (Scrub typhus RCT participant 17, speaking through an interpreter).

Group 2 and 3 respondents were concerned that information could be inadvertently mistranslated and that a great deal of trust was placed on the translators. The general feeling seemed to be that family members were more trustworthy than other sources of informal translators, although they may not have been the most skilled translators. Using translators limited the researchers’ ability to check how much potential research participants understood:

*‘How will we know that it [the information] is covered? It is hard… we don’t understand their language’* (Local doctor and researcher, 26).

The use of informal translators affected information sharing, comprehension and people’s ability to freely choose whether to join research - all essential elements of meaningful consent.

### Decision making to join research: from active decisions to no explicit decisions made

Group 1 respondents’ decision making to join the scrub typhus research ranged from a clear, active decision, to letting researchers carry out the research, to no explicit decision being made. All signed or made their thumbprint on the consent form.

Several Group 1 respondents described making a clear and active decision to join the research study. They gave varying reasons for joining such as finding out what was wrong with them, getting treatment and hoping the research would benefit others in the future. Believing that no harm would come to them was also an important consideration.

*‘I thought about it many times. I decided this project is good. Because it does not cause any damage, it is to help people. I just decided to participate in this project.’* (Scrub typhus RCT participant 11).

Similarly a research nurse described conversations she’d witnessed where patients considered the positives and negatives of joining research:

*‘Their relatives will consult [each other] beside the bed, like, “[shall we] join, [is it] good?”… “There’s nothing bad about it, right? [It’ll] benefit others too” …“Joining is ok”.’* (Research nurse 01, dyadic interview).

The decision was less clear for some, who reported letting the researchers take blood samples or carry out other study processes rather than positively deciding to join the research. Some said they did not think much about joining:

*‘She [participant 17] didn’t think of anything. Because she thought they come to take blood samples and cure her, so she let them do the blood draw… She could do anything if it just could help her get rid of this illness.*

*Interviewer: and who made the decision at that time?*

*Interpreter: She said no one made a decision. They told [her] they would like to take blood samples and [she] let them.’* (Scrub typhus RCT participant 17, speaking through an interpreter).

While some researchers and ethics committee members expressed concerns that people could ‘*participate without saying no’* (Local ethics committee member, doctor and researcher, 32).

For other Group 1 respondents, all of whom belonged to a hill tribe and were reliant on translators, it was less clear that a decision to participate had been made by the participant themselves. Several described being told by their informal translator to make their thumbprint on the consent form with little explanation of what this meant. At other points in the interview they describe being told about the study and various processes that would take place but it was unclear how much they understood when they ‘gave’ consent.

*‘[Her sister-in-law and translator] didn’t say anything. They told her to just do the thumb print, so, she [participant 14] then did the thumb print.* (Scrub typhus RCT participant 14, speaking through an interpreter).

However, they did not express regret at participating in the study. One mentioned that she would recommend that others joined the study and others were simply happy that they were cured from scrub typhus.

### Factors influencing voluntariness of consent

In addition to the social influences of participants’ family members and informal translators described above, the degree to which participants were able to make a voluntary decision to join the research was influenced by other factors, such as, the perceived study benefits, particularly medical care, and burdens on participants. Cultural factors such as respect for and trust in healthcare workers also had an impact, as did the impact of the research on the family unit and wider community. Researchers and ethics committee members were aware of these influences and for some ensuring that participants joined studies voluntarily was their main ethical concern.

*‘But what is worrying, concerning [me], is when researchers go to talk with them, asking [them] to join. Do they spend enough time or provide ample opportunity for asking questions before they sign…because people in general perceive that hospitals are supporters. Consequently, if a doctor makes a request, [you] shouldn’t refuse… should help them. The phrase ‘should help them’ – it might be the first ranked motivation which creates a tendency to join a project out of being ‘kreng-jai’ [considerate], and without reading [and] learning enough details.’* (Local ethics committee member, doctor and researcher, 32).

Despite all Thai citizens and the majority of our Group 1 respondents being entitled to free healthcare regardless of research participation, treatment was seen as one of the main research benefits in both the RCT and observational study and appeared to be a motivating factor for agreeing to join the studies [6]. One participant, for instance, was motivated to join the study so that she would know what was wrong with her and receive the appropriate treatment. This may be a reflection of the broader structural challenges associated with gaining access to healthcare for our study population in this context. The association with research participation and getting the right treatment for their illness could also indicate therapeutic misconception, although there was real overlap between the treatment and research, especially in the RCT. There were also thought to be concerns amongst potential study participants that their care would be affected if they refused to join:

*‘For Thai people, I think it is difficult for them to say no. They come in to get hospital services and we invite them to join the projects. They usually are not brave [enough] to refuse. They may feel that if they decline, it may affect their treatment and care… Whether they are willing to join or not, they will join anyway.’* (Local ethics committee member, doctor and researcher, 08).

A few researchers were concerned that financial compensation could affect people’s voluntariness and unduly influence their decision making if it was high and that the poor would be particularly susceptible to this:

*‘Because if …the compensation is quite a lot of money, then it will be like, unethical. It’s like buying patients into research. Therefore, the compensation has to not be too much… Because it can convey the caretaker’s [parent’s or guardian’s] mind, change the caretaker’s mind to enter the study.’* (Local doctor and researcher 26).

In contrast, most Group 1 respondents viewed the compensation as a positive feature but not something that influenced their decision to join the research.

In addition to the benefits, the perception that the research would have no negative impacts on participants influenced some’s decision making:

*‘She [research nurse] asked me and [she] would like me to join…to participate in the research study like this, was I ok? I then said, “Yes.” Well, there was no [negative] impact to me anyway, instead it gave good benefits*.’ (Scrub typhus RCT participant 21).

Cultural factors had a role to play in people’s decision making to join research. In Thailand, people, including those from the hill tribes, tend to hold healthcare workers in high regard and often feel obliged to help them. Many hill tribe groups previously lacked access to healthcare available to Thai citizens but some medical needs were met by medical missionaries. These medical ‘pioneers’ are still held in high regards by the populations they once served. Healthcare workers, ethics committee members and researchers were concerned that the Thai culture of ‘*kreng-jai’*, which can be defined as being considerate, respectful, showing deference and not wanting to hurt others’ feelings [29] could potentially cause patients to take part in research in order to help and show respect towards the researcher rather than out of their own desire to participate.

*‘Kreng-jai’ [consideration] for the doctors, ‘Kreng-jai’ [consideration] for the nurses. They need it, we lose nothing, no good or bad impacts for us, let’s give them… Asking whether they truly understand? It is not, because [they’re] ‘kreng-jai’ [considerate towards] us. When the doctor asks for cooperation [help], [they’d say] ‘aow-aow’ [meaning ok or agree].’* (Healthcare worker 27).

All groups of respondents thought trust in doctors and healthcare workers, and the high regard they are held with influenced people’s decision making. Some Group 1 respondents described simply following doctors’ advice to join:

*‘The doctor said, participate in the project like this and this, and so I signed, like that.’* (Mother of scrub typhus RCT participant 12).

Despite most respondents feeling that the majority of research participants were able to make a voluntary decision to join the research, a doctor and ethics committee member was worried that healthcare workers or researchers approaching prospective participants in person could pressurise patients to join the research. This could be of concern when participants’ understanding is limited and they rely upon trust or relationships with the healthcare workers to guide their decisions, as described by a research nurse. Although some would argue that basing decisions on trust and relationships is a reasonable approach to decision making.

*‘[Some] do not understand but make the decision right away due to the familiar relationships, and knowing us well, and trusting to an extent. They would give consent right away.’* (Research nurse 23).

### Suggestions for achieving more meaningful consent

#### Increasing research understanding

The importance of understanding research was raised by researchers and Group 1 respondents alike, with the onus being on researchers to ensure participants adequately understood the study in question to make a decision. On the whole, Group 1 respondents did not make many suggestions about improving their research experience but several said they would like to have a formal interpreter. This would help to ensure that all the pertinent information was given and should help to improve understanding of the study and what was meant by the consent process.

*‘She [participant 14] said she wants someone to translate for her. She is unable to speak [Thai]. It was like she didn’t understand what the doctor said’* (Scrub typhus RCT participant 14, speaking through an interpreter).

Another Group 1 respondent commented that she was drowsy while in the hospital so having someone there to help and translate for her would be beneficial. A village chief explained how it was important to have someone from the community translate, who knows the area and has a good heart. A lady who acted as a translator explained that in addition to translating she was able to talk with the participants and help them feel at ease.

Other suggestions for improving individuals’ understanding were taking the time, giving clear explanations and talking nicely:

*‘What [the researchers have] done so far is good already. [Just] ask that we speak nicely to one another, talk together [and] understand, must talk together until [we] understand, right?’* (Scrub typhus RCT participant 03).

The information shared should be given in small chunks, step-by-step, especially if a translator was being used:

*‘May be write them in blocks. Like dolls blocks [small sections at a time]. Simple like this. What to be used here. After that, what will be done. Explain so they understand. The last step is like this. Explain from beginning to the end so they see clear picture about research study from here to here, end here.’* (Healthcare worker 15).

Community engagement was another suggestion for improving understanding. A village chief suggested running community events to exchange ideas and increase understanding and a researcher suggesting bringing in previous research participants so that they can share their experiences.

*‘We must organise a training or seminar, [that’ll] also be good, for exchanging [ideas]; set up a stage if [we] want [it] to be effective [laughs].’* (Village chief 24).

Involving people’s support networks was recommended, reflecting the importance of the collective as well as the individual’s influence on decision making. A Group 1 respondent said researchers should include her relative as she would be able to understand and help discuss with her. A research nurse noted how participants are usually recruited in the hospital setting away from their community and normal support networks. Despite people having autonomy to decide alone she wanted the community to be involved. More widely improving education, Thai language and the general public’s understanding of health and research could help to improve individuals’ understanding.

#### Ensuring voluntary decision making

The main suggestions to enable participants to join or choose not to join voluntarily were to explain that this was their decision and to make sure they understood the study. It was generally thought that witnesses helped to protect participants. Other suggestions included asking patients to come back another day if they wanted to participate and advertising studies on posters rather than approaching patients directly:

*‘If put up posters to invite them, they have freedom to make decision to join or not join. But if we [researchers/healthcare workers] approach them personally… it’s like… they are ‘kreng-jai’ [considerate], they don’t dare to refuse.’* (Local ethics committee member, doctor and researcher 08).

To prevent compensation from unduly influencing the potential participants it was typically discussed towards the end of the consent process. A Group 1 respondent also noted the need to compensate family members’ time if they were needed to facilitate follow-up visits.

## Discussion

Meaningful valid consent is a foundational ethical principle required for research participation, yet achieving meaningful consent is challenging [11–13]. Research ethics guidelines detail the information which should be given and understood by prospective participants, yet the importance of each item of information can vary for different individuals [10, 14, 27, 30, 31].

In northern Thailand, working predominantly with hill tribe groups we found that participants’ were provided with so much information that they could not remember, and understanding of the research varied greatly. Several Group 1 respondents showed little understanding and did not describe a clear decision to join the research study; they instead followed the instructions or advice of the person translating for them and put their thumbprint on the consent form. This mixed understanding did not seem to be influenced by the type of study participants were enrolled in (RCT or observational). Research participants from varied settings have often been found to have limited understanding of research; this challenge is present globally and is not limited to lower resource settings [12, 13, 27, 32]. In addition, individuals will require varying amounts of information and levels of understanding in order to come to a decision. What is adequate understanding to one may not be to another. Importantly, factual knowledge about research does not always equate to understanding and understanding may not be the main basis of people’s decision making [33]. Millum and Bromwich argue that full understanding of research, the potential risks, benefits and experiences participants will have, is not possible. Instead, participants need to understand what giving consent means, that they can decline and what they are consenting to - which is the most challenging part to achieve [34].

Informal translators provided an important, but imperfect, source of support for non-Thai speaking participants, enabling them to understand more about their illness and the opportunity to join the research. This role is particularly important in an area where multiple minority languages are spoken and the use of informal translators is common. Translators (both formal and informal) can act as advocates for participants, informal translators in particular see their role as supporting and advising people in addition to translating [35, 36]. Informal translators have reported that perceived time pressures mean they summarise, omit or translate information later [35, 36]. In order to try and increase their clients’ understanding interpreters can add, subtract and alter the language and information given as well as emphasising or down playing points [36]. At times we found the translation of study information was inadequate and added to the challenges of ensuring participants had sufficient understanding of the research. Given the difficulties of achieving adequate understanding when speaking the same language it is unsurprising that more challenges will present when working through a translator and that informal translators will make more errors than formal translators [37, 38]. Similar to other settings, understanding could also be affected by the sheer volume of information shared, unfamiliarity with the disease and research, and the fact that many local languages do not have a direct translation for the word ‘research’ [11, 12].

Despite the barriers to understanding, it is important to highlight our interviewees’ report of trial participants’ agency in decision making, even if constrained [39]. The degree of decision making ranged from an active decision to no clear decision being made, and deference to others. People’s decisions were not made in isolation but were influenced by social, cultural and economic factors. Decision making was influenced to a greater or lesser extent by family members, translators and healthcare workers. Trust in others and cultural attributes like ‘*kreng-jai’* led to some joining the research [12, 40, 41]. The cultural attribute of ‘*kreng-jai*’ is similar to the need to be polite and not refuse requests from outsiders seen in diverse settings including Kenya and India [11]. This is in and of itself not wrong; Bull and Lindegger describe how the influence others have on the decision to participate in research can range from independent voluntariness, where the participant decides but is influenced by others, to co-operative decision making, to a controlled decision which is effectively proxy consent [16]. Similarly, Ngure et al. have described the importance of recognizing the social nature of decisions to join research in the Kenyan context, offering a more relational account of promoting participants’ autonomy [42]. The collectivist culture which predominates in Thailand over individualism means that value is given to doing good for others; this social, collective view can compete against individuals’ autonomy. This could be represented by participating in research to help healthcare workers or for the future benefit of others. The challenge is ensuring that all participants but especially those that are reliant on others to participate are enabled to make an independent or co-operative decision to join research.

Several prospective respondents did decline to join this qualitative study, the majority of these said it was due to time constraints. However, one lady agreed to an interview but while going through the study details on the interview day it became clear that she was uncomfortable and appeared not to want to take part. Yet despite explaining to her that the decision was hers and that she should not feel any obligation to take part or feel ‘*kreng-jai*’ towards us she did not say no to an interview. Instead she said she would do it another day. We gave her our contact details and asked her to call us if she wanted to do the interview. We have not heard back from her since. Similar cases of ‘silent refusals’ have been described in the literature where it can be unclear if people want to join or continue to participate in research studies [43].

Even modest compensation or provision of health services strongly influence decisions to join research, a finding consistent with the literature from low-and-middle income countries where healthcare is seen as one of the main reasons to join studies [12, 15, 27, 44]. When research occurs in contexts of deprivation, these structural factors need to be considered in the study design and provision of fair but non-coercive benefits. Having a choice alone is not enough; researchers need to ensure the quality of options available to participants; an ‘empty choice’ is not sufficient [15]. Kingori describes how people’s choices to join research studies can be heavily influenced by structural and contextual factors, such as a lack of alternative options for accessing healthcare. This can mean that despite have a choice to join research it is effectively an ‘empty choice’ or no choice at all [15].

Our study offers a more holistic picture of the consent process from a range of respondents, providing different perspectives on hospital-based research in northern Thailand. The use of interpreters allowed us to include and explore the experiences of hill tribe people who cannot speak Thai, an important, under-represented group in this area. However, despite training our interpreters we were still limited by their use in the interviews which heightened the challenges of exploring participants’ understanding of the clinical studies, as did the lack of word for ‘research’ in Akha and Lahu. There was a time delay between Group 1 respondents giving consent to join the clinical study and our interviews, in part to ensure recovery from their illness which may have affected their recall of events and led us to underestimate their understanding of the consent process and research.

We expect that many of the challenges found in this study will resonate with researchers working in other settings, especially the challenges of effective communication about research and attaining adequate research understanding. Potential solutions and learning should be shared globally and adapted to suit different contexts. Researchers need to be aware that some participants will join studies even with limited research understanding and despite having uncertainties about the research, others may not even be aware they have joined a research study. Research institutions need to work alongside participants and with the community to increase participants’ understanding of consent and what it will mean for them. Community engagement programmes can be used to increase the community’s research understanding rather than placing the burden purely on individual participants and researchers during recruitment. Examples of this work include working with community advisory boards or community representatives [45, 46]. Further work is being done locally with the community to co-create materials to be used in the informed consent process and to develop explanations for common research terminology. Careful planning and consideration of the language, style and volume of information given to all participants is needed. Additionally, in response to the challenges around translation, during our data collection period we established a network of formal interpreters who were able to support patients throughout the informed consent process. Training was provided to ensure that all the pertinent information was shared enabling patients to make decisions for themselves. Where translation is required back translation should be used to ensure that the key messages are conveyed adequately and clearly. Researchers should be trained to work through interpreters where relevant. Evaluation is required to see if these measures are improving participants’ research understanding and making the informed consent process more meaningful.

Local understanding of how people make decisions, and the social and cultural factors that can influence them should be considered when planning research studies and taking informed consent. The power dynamics at play when healthcare workers and researchers, especially those from different cultures and socio-economic statuses ask patients to participate in research need to be taken into account. Ultimately health inequalities and power imbalances need addressing in order to allow true voluntariness. Research is needed as to how to enable participants to make a more active decision about research participation.

## Conclusions

Achieving meaningful consent is challenging but is a key ethical responsibility for researchers. Information about the research needs to be communicated to prospective participants in a way that they can understand to help support their decision making. Decision making to join research is not based purely on research understanding but will be influenced by perceived study benefits and burdens, as well as social and cultural factors.

## Data Availability

The datasets generated and/or analysed during the current study are not publicly available due to ethical and legal reasons, including participant anonymity but are available from the MORU Data Access Committee on reasonable request. A data access agreement will be put in place prior to data transfer. Instructions and the data application form are available here: https://www.tropmedres.ac/units/moru-bangkok/bioethics-engagement/data-sharing.

## List of abbreviations

MORU: Mahidol Oxford Tropical Medicine Research Unit
RCT: randomised controlled trial
REACH study: Resilience, Empowerment and Advocacy in Women’s and Children’s Health
